# The Effects of a Humanoid Socially Assistive Robot Versus Tablet Training on Psychosocial and Physical Outcomes of Persons With Dementia: Protocol for a Mixed Methods Study

**DOI:** 10.2196/14927

**Published:** 2020-02-05

**Authors:** Sandra Schüssler, Julia Zuschnegg, Lucas Paletta, Maria Fellner, Gerald Lodron, Josef Steiner, Sandra Pansy-Resch, Lara Lammer, Dimitrios Prodromou, Sebastian Brunsch, Magdalena Holter, Lorenzo Carnevale, Silvia Russegger

**Affiliations:** 1 Institute of Nursing Science Medical University of Graz Graz Austria; 2 Institut Digital Joanneum Research Forschungsgesellschaft mbH Graz Austria; 3 Sozialverein Deutschlandsberg Deutschlandsberg Austria; 4 Humanizing Technologies GmbH Vienna Austria; 5 Institute for Medical Informatics, Statistics and Documentation Medical University of Graz Graz Austria

**Keywords:** dementia, socially assistive robot, home care, caregiver, dementia trainers, motivation, physical training, cognitive training, care burden, humanoid robot

## Abstract

**Background:**

New technologies, like socially assistive robots (SARs), may have the potential to support caregivers at home. Still, the evidence for people with dementia in home care is unclear because a lot of studies are performed in a laboratory or institutional setting, and mainly use robots in prototype stages.

**Objective:**

This study aims to explore the effects of the refined, commercially-available, humanoid SAR Pepper combined with a tablet PC–based dementia training program (Coach Pepper) versus an exclusively tablet PC–based dementia training program on psychosocial and physical outcomes of people with dementia living at home, including caregivers and dementia trainers. We hypothesize that Coach Pepper has a more positive effect on the primary outcome motivation (stable or decreased apathy) of people with dementia.

**Methods:**

A mixed methods study will be performed, including a randomized controlled, parallel, 2-arm study with a complementary qualitative part. This sample includes 40 PWD living at home and 40 relatives, each complemented with five professional caregivers and dementia trainers. The intervention group will receive Coach Pepper (a SAR connected with a tablet PC–based dementia training program), and the control group will receive exclusively tablet PC–based training without the SAR. The duration of the intervention will be three weeks per household. Data will be collected at baseline and during and after the intervention by standardized questionnaires, sensor data of the robot, and tablet PC, as well as semistructured interviews, focus groups, and observation.

**Results:**

To date, no results are available for this study protocol. The study intervention started in May 2019 and will end in Spring 2020.

**Conclusions:**

The intervention of this study can be seen as a nonpharmacological intervention, including cognitive and physical training by a robot. This study will help to further refine SAR for the specific needs of people with dementia living at home.

**International Registered Report Identifier (IRRID):**

DERR1-10.2196/14927

## Introduction

### Background

Dementia rates are increasing worldwide and consequently burden global health care resources to a serious degree [1,2]. On the other hand, there is a decreasing number of available caregivers to provide (nursing) care [3-5]. (Nursing) care of people with dementia usually takes place at home, especially in the early stages [6]. Owing to the progression of dementia and growing (nursing) care needs because of increasing care dependency (eg, in mobility, social contacts, and learning ability), (nursing) care problems (eg, incontinence and malnutrition), professional care (eg, by nurses) and a possible nursing home transition become increasingly necessary [6-8]. One of the most important aims in (nursing) care for people with dementia is to promote their independence according to their stage of dementia and individual abilities. Such (nursing) care can counteract a galloping progression of care dependency [9]. It is in this context that new technologies, such as socially assistive robots (SARs), may constitute a supportive device for caregivers because they have the potential to promote the independence and well-being of older people [10,11].

SARs can be defined as representing an intersection of assistive robots (giving aid or support to a human user) and socially interactive robots (social interaction through speech and gestures) [12]. The goal of SARs is to create a close and effective interaction with a human user by giving assistance through social interaction (eg, in activities of daily life ranging from cognitive to physical tasks or to encourage emotional expression, conversation, and gestures) [12]. The appearance of the SARs can vary. They can look like a mechanoid with a machine-like appearance, or like a humanoid, such as Pepper by Soft Bank Robotics, which is designed with an unrealistic but still human-like appearance so that users can identify it as a robot. They can also look like an android with an almost realistic human-like appearance, or, in the case of animal-like SAR, can look like an animal, such as Paro, the seal baby [13,14]. In this study, the humanoid SAR Pepper (see section Interventions) is used.

Results of various reviews (literature, scoping, and systematic) show that research on SAR in the context of older people with and without dementia is most often conducted on SAR with an animal-like appearance, such as Paro, which was designed with the appearance and behavior of a baby seal, AIBO, the robotic dog, or NeCoRo, the robotic cat [13,15]. However, there is a wealth of studies relating to other robot types, like humanoid SAR [15,16].

Until now, the effectiveness of SAR in all care settings, especially in-home care, has generally been unspecified. Studies show that these robots may have a positive impact on affect, cognition, physiological parameters, use of medications, social contacts, and quality of life with respect to well-being and behavior [13,15,16]. Regarding behavior, apathy, which is defined as a loss of motivation [17], is one of the most common behavioral and psychological symptoms (BPSD) in people with dementia, with an overall mean prevalence of 49% [18].

**Figure 1:**
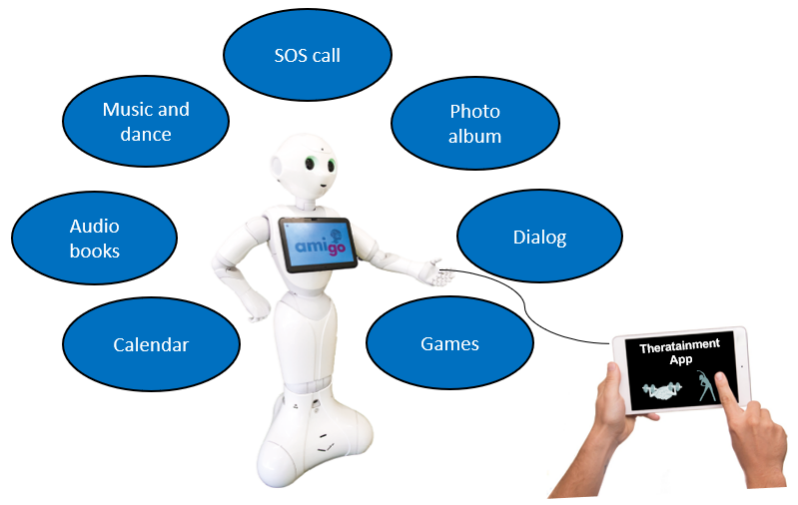
The functions of Coach Pepper.

Measurements from research studies have demonstrated that people with dementia have lower capacities for motivation processes [19]. Current models of motivation identify and discriminate two phases: (1) goal setting; and (2) goal pursuit. The latter requires self-regulatory capacities for decision-making, regulation of activation, and regulation of motivation. Forstmeier and Maercker [19] concluded from their research that cognitive and physical training should be complemented by motivation-supporting training strategies, such as goal-setting and self-motivation [19]. In addition, motivation-oriented interventions support the reduction of neuropsychiatric symptoms, such as depression and apathy (loss of motivation). The study described in this protocol implemented motivation-oriented strategies into the overall technological Coach Pepper concept in the shape of a humanoid SAR, which worked to motivate the people with dementia by means of social interaction to perform daily dementia training on a tablet PC (all functions of Coach Pepper are shown in Figure 1).

It is necessary to focus on the motivation of people with dementia, because loss of motivation (apathy) could entail a decline in cognition, problems in activities of daily living (ADL), decreased quality of life, increased morbidity, greater mortality, and for caregivers, a greater caregiver burden [20-22]. In a systematic review by Pu et al [15], only two studies were found which included apathy as an outcome measure of SAR interventions. Only one of these studies included a humanoid SAR as an intervention for people with dementia. None of these two studies were performed in home care, which is a setting where a lack of research with SAR is still prevalent.

In their scoping review, Buhtz et al [23] identified 19 studies that included SAR for care-dependent people at home. Most of these robots were in a prototype stage and were tested mainly for technical aspects and operability in predominantly exploratory or piloting studies. Thus, there is a recommendation to explore the effectiveness of SAR [15,23] in well-designed randomized controlled trials with larger sample sizes [23,24]. In home care, often no more than ten older people with or without dementia are included in studies using SAR [25-28]. This is not surprising, because the home care setting can be seen both in technical and scientific terms as one of the most challenging and complex scenarios for SAR. A household with people and objects that seemingly unpredictably vary their position presents SAR with enormous challenges and hurdles [23]. But research of SAR in home care is extremely important, because many older people with and without dementia would like to live at home as long as possible. SAR, as an innovative intervention, has the potential to support care independency (in various ADL) at home and may help to avoid or delay institutional care (eg, nursing homes).

### Overall Aim

The overall aim is to explore the effects of a humanoid SAR versus an exclusively tablet PC–based dementia training on psychosocial and physical outcomes of people with dementia living at home, including caregivers and dementia trainers.

#### Primary Aims

The primary aims of this study include exploring the effect of Coach Robot Pepper on motivation (in the sense of increased, decreased, or stable apathy) of people with dementia versus the tablet PC–based training, and exploring the effect of Coach Robot Pepper on the care burden of relatives compared with the tablet PC–based training.

#### Secondary Aims

There are several secondaru aims of this study, one of which is exploring the effect of Coach Robot Pepper on acceptance, usability, quality of life, cognition, mobility, depression, behavioral problems, and care dependency of people with dementia versus the tablet PC–based training. We would also like to explore the effect of Coach Robot Pepper on depression, quality of life, affect and acceptance, and usability of relatives versus the tablet PC–based training. There will also be a supplementary investigation of the acceptance and usability of robot Pepper and the tablet PC–based training in dementia trainers and caregivers, a supplementary observation of people with dementia to get an insight into how to handle a robot and the tablet PC–based training in home care (including usability), and supplementary interviews (focus groups or individual interviews) to obtain a deeper understanding of the experience (including usability) of all participants using Coach Robot Pepper in home care.

The description of the study protocol follows the Standard Protocol Items: Recommendations for Interventional Trials guideline [29].

## Methods

### Design

A mixed method study with embedded design will be performed. The quantitative part will be a randomized, controlled, parallel, two-arm study, and the complementary qualitative part will include semistructured, guideline-based interviews. This design was chosen to not only obtain quantitative results but also to get a deeper insight into the experiences of using a SAR in home care for people with dementia.

### Setting and Sample

#### Setting

This study will be performed in the private households of people with dementia living in Styria, which is one of the 9 federal states of Austria, with 1,239,153 inhabitants [30] in 540,800 private households and an average household size of 2.25 people [31].

#### Sample

We will include people with mild and moderate dementia as the main sample. We will also include their main relative as well as their nursing staff (nurses and nursing assistant) and dementia trainers. The inclusion criteria are presented in Textbox 1.

**Inclusion criteria.**

**Persons with dementia**

- adults
- living at home
- all types of dementia (except frontotemporal dementia)
- mild or moderate dementia (Mini Mental State Examination 10 and above)
- mild dementia: living alone or with a relative at home (if alone, the relative should live in the neighborhood and be in daily contact with the person with dementia)
- moderate dementia: living with a relative at home
- receive professional or nonprofessional care or no care
- speak and understand German
- have no physical, auditory, or visual restrictions, which would make the application of the interventions impossible
- do not take any dementia-specific medication or have been taking dementia-specific medication for at least 3 months; condition stable and no change expected during the study period
- do not take antipsychotics and antidepressants or have been taking them for at least 14 days before study start
- children and pets in the household after previous individual discussion

**Relatives**

- relatives of the participating people with dementia (adults)
- living or not living with the person with dementia in the same household (in the case of moderate dementia, relatives must live in the same household)
- person with dementia receives or does not receive professional care
- relative provides or does not provide care
- if the people with dementia receive paid 24-hour care (regardless of whether they have mild or moderate dementia), a relative still has to be recruited as a participant (this relative must live in the same house or household and be in daily contact with the person with dementia)
- speak and understand German

**Nursing staff**

- adults
- nurses or nursing assistants
- speak and understand German

**Dementia trainers**

- adults
- trained as Morbus Alzheimer Syndrome trainer
- train the participants with dementia at home
- speak and understand German

### Sample Size

There are currently no comparative studies investigating the influence of humanoid robots on the motivation of people with dementia. Therefore, no results can be used to calculate an optimal sample size. However, to gain insight about what is feasible with our sample size, we estimated the possible effect size. For simplicity, sample size considerations are based on a Student’s *t* test. A sample size of 20 in each group will have 80% power to detect an effect size of 0.91 using a two-group *t* test, with a 5% two-sided significance level. For example, if there is a mean difference of 10 between the groups and a standard deviation of 11 (the latter being assumable according to the literature [32]), the effect size would be 0.91. As a drop-out rate of 20% to 30% can be assumed, 20 people with dementia per group was planned (40 in total). This is the maximum number of people that can be realized with two robots during the study period of 10 months. Beside the people with dementia, their main relatives will be included (n=40, 20 each group) and as a supplement five nurses, nursing assistants, and dementia trainers, respectively, will be included. A small sample size of nursing staff and dementia trainers were chosen because a minimum of three people are necessary for usability tests (which is one focus in the study), if there are more than three groups included [33]. The inclusion of these further two groups was necessary to get an in-depth understanding of using robots in home care.

### Recruitment

The recruitment of the participants will be carried out by project members of a social nonprofit organization in the community. The sampling method will be convenience sampling. This organization runs the first dementia service center in Styria and offers advice and consultation for relatives providing care at home. They also offer Morbus Alzheimer Syndrome training in private households. All potential participants will be contacted personally or by telephone by the nonprofit organization. Interested participants will be offered a home visit to inform them in detail about the study by means of an information folder (including detailed information about the study) and a short video about the robot Pepper. For the recruitment, flyers will be placed at the service points of the social nonprofit organization, at the organization’s regional events, in waiting rooms of medical practices, and on social media platforms. Only participants who were willing to be assigned to either the intervention or control group were included.

### Randomization and Blinding

A randomization plan will be prepared by the Institute of Medical Informatics, Statistics and Documentation at the Medical University of Graz. For that, randomization software will be used. Only authorized people will be able to randomize patients, and the allocation to the intervention and control group will be balanced. There are two robots available for simultaneous use in the study, therefore, four people will always be randomized (two for intervention and two for the control group) two weeks before the next round starts. This will be done until all 40 people have been randomized. A single blinding will be performed. The clinical health care psychologist who will perform data collection before and after the interventions will be blinded.

### Ethics Approval and Consent to Participate

This study follows the Declaration of Helsinki and received ethical approval from the Ethics Committee of the Medical University of Graz, Austria (Approval Number: 30-401ex17/18). For all participants, written informed consent by project members of the social nonprofit organization will be obtained. If people with dementia have a legal representative, the written informed consent will be given by them. If a person with dementia is not able to give written informed consent by themselves and has no legal representative, he or she will be not included in the study. Every person with dementia and all robots will be insured during the study. The participants can drop out of the study at any time, and in the event of health hazards, the study will be stopped immediately for the affected person.

### Interventions

#### Robot Pepper

Pepper is a humanoid SAR from the company SoftBank Robotics. Pepper is 1.20 m tall and weighs 28 kg. Pepper has four microphones, two high definition cameras, and a depth-perceiving sensor that gives Pepper three-dimensional sight of his surroundings. Pepper talks in different languages like English, French, and German, and it has a touchscreen tablet on its torso. An internal gyro sensor gives Pepper information about the position of its body. Pepper can make fluid and expressive movements with its arms, and while the hands are equipped with touch sensors, Pepper is unable to pick up objects. Furthermore, Pepper has 3 bumper sensors and laser sensors as well as sonars to estimate distances to obstacles. Omnidirectional wheels enable Pepper to move and rotate on the spot. Robot Pepper is not able to navigate in rooms because of software restrictions. Peppers’ operation time is about 12 hours. For this study, the functions of Pepper were refined according to the results of a prior qualitative study with the aim of exploring the needs of people with dementia and a follow-up pilot study where the first refined prototype was tested (mainly for acceptance regarding the robot’s usability). The functions of the refined Robot Pepper can be seen in Figure 1.

#### Tablet PC–Based Dementia Training

The training program was developed in a prior study for people with dementia living at home or in institutional care. The training includes a serious game with a cognitive and physical training program, and the training can be tailored to an individual (eg, content, level of difficulty adapted to the stage of dementia, procedure, and time). The training always starts first with physical exercises (eg, balance, motor skills, and coordination), which are explained by text and video on a tablet PC. This is followed by cognitive exercises, including quizzes, spot-the-difference puzzles, puzzles, looking for picture pairs, cloze tests, mathematical tasks, listening tasks, and songs.

#### Intervention Group (Coach Pepper)

For the intervention group, the robot Pepper is virtually connected via Web interfaces with the dementia training program on an additional tablet PC. Therefore, the intervention group is called a Coach Pepper group (Figure 1).

The total study duration is 10 months (three weeks per household). The planned start is May 2019. Because there are two Pepper robots in the study, the robot is transported from the first two private households to the next two private households. This means that the intervention starts with the first two people with dementia, who receive Coach Pepper for three weeks. Thereafter, there is a break of one week when the training and individual adaptation of the robot for the next two households takes place. After this, the next two people with dementia receive the intervention for three weeks. This happens until all 20 people with dementia have received their interventions. Table 1 shows the time schedule of the study.

**Table 1:** Time schedule: an example of two study rounds.

Study phases		Enrollment (Ongoing)	Randomization/allocation (2 weeks before intervention)	(Break)	Study round 1			(Break)	Study round 2			(Break)
				Week 0	Week 1	Week 2	Week 3	Week 0	Week 1	Week 2	Week 3	Week 0
**Enrollment**												
	Eligibility screen	✓^a^	—^b^	—	—	—	—	—	—	—	—	—
	Informed consent	✓	—	—	—	—	—	—	—	—	—	—
	Randomization/allocation	—	✓	—	—	—	✓	—	—	—	✓	—
**Interventions**												
	Coach Pepper	—	—	—	✓	✓	✓	—	✓	✓	✓	—
	Tablet PC–based dementia training	—	—	—	✓	✓	✓	—	✓	✓	✓	—
**Assessments**												
	Questionnaires	✓	—	✓	—	—	—	✓	—	—	—	✓
	Sensor data	—	—	—	✓	✓	✓	—	✓	✓	✓	—
	Observation	—	—	—	✓	—	✓	—	✓	—	✓	—
	Interviews, focus groups	—	—	—	—	—	—	✓	—	—	—	✓

^a^Time of enrollment, intervention, and assessment tasks.

^b^Not applicable.

Due to robot Pepper's restricted mobility (navigation is not possible), the robot will stand on a previously defined place in the household (eg, living room) where the person with dementia spends most of the day. Pepper will start communication proactively when the user is in proximity (person in proximity recognition and proactive dialogs) or by a date and time previously entered (time-triggered proactive dialogs). Pepper will encourage the people with dementia to use the tablet PC–based dementia training, and will guide them through the training with speech, gestures, music, and dance. The physical exercise videos as well as the correct answers for the cognitive exercises will be displayed on robot Pepper’s Tablet. Furthermore, Pepper will motivate the participants to use further functions of Pepper itself. However, it is also possible for people with dementia or their relatives to start Coach Pepper at any time.

During the test phase, a dementia trainer comes to the household once a week for one hour to perform the dementia training together with the people with dementia. A nurse or nursing assistant will also come as a visitor in the first and last week of the test period to perform a one-hour observation of the people with dementia. Outside of these times, the participants can independently use Coach Pepper. In addition, all households will receive regular calls from the research team to discuss questions or issues. For all participants, measurements are taken before, during, and after the intervention period (see Multimedia Appendix 1).

#### Control Group (Tablet PC–Based Training)

The control group will exclusively receive the tablet PC–based dementia training, without the robot. Otherwise, it will be the same procedure as in the intervention group. In total, two people with dementia start the intervention for three weeks, and there will be one week’s break with training for, and adaptations of, the tablet PC–based dementia training for the next two people. Then, a three week test period will happen again with the next two people. This will happen until all 20 people have received the control intervention. Just as in the intervention group, a dementia trainer comes to the household once a week for one hour and a professional caregiver comes twice (first and last week of testing) for observation. Outside these times, people with dementia and their relatives can use the tablet PC training as often as they like. Regular control calls will also be made. For participants in the intervention and control group, it is forbidden during the study to use (similar) devices (eg, robots, tablets, and smartphones) including any cognitive or physical training.

### Training

#### Pepper Master Training

Before the intervention, all responsible project members (eg, dementia trainers, project assistants, and technical people) will receive Pepper Master Training, which will be carried out by the project partner Humanizing Technologies. In this training, Pepper will be presented with its functionalities, including how to unpack, put into operation, and repack the robot. Important notes about the system, daily use, maintenance, and troubleshooting will also also discussed. The training will last two hours. The trained people will be responsible over the course of the project to handle questions/problems from the participants with regard to robot Pepper.

#### Training Data Collection Methods, Course of Study

To ensure consistent data collection, all data-collecting people will be trained by the researchers of the Institute for Nursing Science regarding the course of study, as well as all data collection methods. The duration will be about four hours.

#### Intervention Training

The social nonprofit organization will train their dementia trainers and nursing staff on the interventions with the tablet PC–based training and Coach Pepper. The duration will be about four hours. They will also train the people with dementia and their relatives. The training will be in the private household of the participants on the day of delivery of Coach Pepper or the exclusively tablet PC–based training (first day of intervention, always on Monday). The duration of the training depends on the individual needs of the participants. Every household will receive an operating manual for Coach Pepper and the tablet PC–based training program.

During the study, a hotline will be set up for participants’ questions and problems. Home visits will be offered if problems cannot be solved on the telephone. Furthermore, control calls will be performed regularly by project members to ensure that participants can handle Coach Pepper and the tablet PC–based training program.

### Measurements

An overview of the following data collection methods is outlined in Multimedia Appendix 1. If a participant drops out after randomization, the minimum amount of data collected by this person are the sample characteristics (eg, age, gender, and education). All important changes of measurement methods will be indicated in the trial register. The measurements are shown in Multimedia Appendix 1.

#### Primary Outcome Measurements

##### Motivation

The Apathy Evaluation Scale (AES) will be used to measure motivation because apathy can be understood as a loss of motivation. The scale has 18 items (4-point Likert scale), and a total of 18-72 points can be obtained. Higher scores correspond to a higher degree of apathy, and therefore lower motivation [34,35].

##### Care Burden

The Zarit Burden Interview will be used to measure the subjective care burden of the relatives. The instrument has 22 items (5-point Likert scale), and a total of 0-88 points can be obtained. Higher scores indicate greater caregiver distress [36-38].

#### Secondary Outcome Measurements

##### Quantitative Measurements

###### Quality of Life

The Dementia Quality of Life Questionnaire will be used to measure the health-related quality of life of the people with dementia. The questionnaire consists of a self-rating version for people with dementia with 28 items and a proxy version for their relatives with 31 items. Each version also has an additional item to capture the global quality of life of the person with dementia [39,40]. Both versions are applied during an interview, thus capturing the emotions, memory, and everyday life of the person with dementia during the last week [39,41]. A 4-point Likert scale is used to collect responses and a higher overall total score reflects a better health-related quality of life [38]. For the relatives, the World Health Organization Quality of Life Scale-BREF will be used. It has 26 items and 4 domains (physical health, psychological, social relationship, and environment). For every item, 1-5 points can be obtained. In general, higher domain scores indicate a higher quality of life [42,43].

###### Care Dependency

The Care Dependency Scale will be used to measure care dependency of people with dementia. The scale has 15 items (5-point Likert scale). In total, 15-75 points can be obtained, and lower scores indicate a higher degree of care dependency [44].

###### Mobility

The Timed UP and GO Test (TUG) will be used to measure mobility in people with dementia. The test measures the time (in seconds) an individual needs to stand up from a standard arm chair, walk a distance of 3 m, turn, walk back to the chair, and sit down. Interpretation: <10 seconds=completely unrestricted; 10-19 seconds=less mobile, but still unrestricted; 20-29 seconds=limited mobility; >30 seconds=pronounced mobility restriction; 14 seconds and more has been shown to indicate a high risk of falls [45].

###### Cognitive State

The Montreal Cognitive Assessment will be used to assess cognition in people with dementia. The instrument has 30 items in 8 domains of cognitive functioning: attention and concentration, executive functions, memory, language, visuoconstructional skills, conceptual thinking, calculations, and orientation. In total, 0-30 points can be obtained, and lower scores indicate a higher degree of cognitive impairment [46,47].

###### Depressive Symptoms

The Geriatric Depression Scale will be used to assess depressive symptoms in people with dementia. The scale has 15 items (yes/no answers). In total, 0-15 points can be obtained, and higher scores indicate a higher level of depressive symptoms [48-50]. For the relatives, the Center for Epidemiological Studies Depression Scale will be used. The scale has 20 items (4-point Likert scale), and a total of 0-60 points can be obtained. Higher scores indicate a higher level of depressive symptoms [51,52].

###### Acceptance and Usability

The Technology Usage Inventory will be used to measure acceptance in all included participants. It captures technology-specific and psychological factors that contribute to the use of a technological device. The instrument includes 8 main dimensions (curiosity: 4 items; anxiety: 4 items; interest: 4 items; usability/user-friendliness: 3 items; immersion: 4 items; utility: 4 items; skepticism: 4 items; and accessibility: 3 items) with 30 items in total (7-point Likert scale). For every dimension, 1-21 or 28 points can be obtained. Furthermore, the instrument includes a ninth dimension (intention to use with 3 items). This ninth dimension is measured on a visual analog scale, including a 10-centimeter long horizontal line with two endpoints (agree and disagree). A cross on the line indicates the degree of agreement. For the evaluation, the distance from the right endpoint (disagreement) to the answer across the line is measured. This distance (in millimeters) is determined and summed up for all 3 items (maximum: 300, minimum: 0). For all 9 dimensions, higher levels on the respective dimension indicate a higher level of expression in the respective construction [53].

###### Affect

The Positive and Negative Affect Schedule will be used to measure effect of the relatives. The instrument has 20 items (5-point Likert scale) with two dimensions (positive affect and negative affect). In total, 20-100 points can be obtained, and higher scores indicate higher positive or rather negative affect [54].

###### Behavioral Problems

The Neuropsychiatric Inventory (NPI) will be used to measure behavioral problems of people with dementia, and information will be obtained from a caregiver who is familiar with the patient’s behavior. The instrument has 12 domains (delusions, agitation/aggression, depression, anxiety, elation/euphoria, apathy/indifference, disinhibition, irritability, aberrant motor behavior, sleep and night-time behavior disorders, appetite, and eating disorders). The NPI assesses the presence, frequency, and severity of each behavior in the previous month, as well as the level of caregiver distress as a result of each of the neuropsychiatric problems. The domain score is obtained by multiplying the frequency and severity scores. The total NPI score is finally the sum of all individual domain scores (thus, ranging from 0-144). The caregiver distress level is not part of the total NPI score. Higher scores indicate greater psychopathology [55,56].

###### Sensor Data

Furthermore, sensor data from the robot platform Pepper and the theratainment app will be continuously collected during the study period. The objective is to extract key features from the sensor data and investigate correlations with the scores from the used questionnaires (AES, Montreal Cognitive Assessment, and TUG) to apply advanced machine learning techniques to research the potential of developing statistics-based estimators to predict motivation, cognitive state, and mobility/physical activity.

##### Qualitative Measurements

Open, semistructured observation of people with dementia interacting with the robot at home will be conducted by professional caregivers to explore how they handle the robot during the study period. Besides individual interviews with the people with dementia and relatives, focus groups will also be organized with the professional caregivers and dementia trainers to obtain more in-depth knowledge about their experience with the robot.

###### Data Management

The sensor data of robot Pepper will be processed directly for the interaction and not be stored. A connection to Pepper from outside (eg, tablet PC–based training program) is only possible via a secure connection and with a user ID or password. During the study, sensor data is immediately forwarded to the appropriate project partners for processing via secure connections. All sensor data are analyzed anonymously and result in anonymized feature data. The videos themselves are deleted immediately after extracting the features. For each completed dementia training exercise, the tablet PC stores data reflecting the performance of each participant with dementia (eg, wrong/correct answers, quizzes, and time). All data will be analyzed anonymized.

The questionnaires and interviews will be handed over by project members of the social nonprofit organization either personally or via a secure server (protected password) to the Medical University. All personal data of participants will be treated confidentially and interviews will be anonymized during transcription. All participants will be assigned a code. The Medical University and Joanneum Research (research partner) have access to the final data set for analysis. To ensure data quality, all data from paper-pencil questionnaires will be entered into the statistical software by one researcher and will be scanned for errors after data entry by the same person. Furthermore, sample checks for data errors by a second researcher are planned, and the statistician will also perform a plausibility check of the data before starting the analysis.

### Analysis

#### Quantitative Analysis

Statistical analyses of the results will be performed following the intention-to-treat principle. Descriptive statistics of the data will be presented as a mean and standard deviation, or median and quartile, depending on the nature of the distribution. To describe categorical data, absolute and relative frequencies will be used. To answer the primary question as to whether the motivation differs between the two intervention groups, a median regression is planned. Therefore, it can be adjusted for the degree of dementia and depression. For differences between the intervention groups for the secondary outcomes, the ordinal scale data will be analyzed with median regression. For metric data, a covariance analysis is planned, which also adjusts to degrees of dementia and depression. Changes in pre- and postintervention outcomes concerning relatives will be analyzed by a paired *t* test or a two-tailed Wilcoxon signed-rank test, depending on the distribution of the data. For the sensor data, correlations with the scores of questionnaires will be performed. Furthermore, a skeleton-based analysis of human activity will be applied on the video frames of the physical exercises [57]. The estimated increase of kinetic energy of the movements is intended to provide cues for the increase of motivation [58]. In addition, the video data will be analyzed for nonverbal expressive features which provide analytics about the state of mobility. The significance level will be set to alpha=0.05. For the evaluation, SAS 9.4 (SAS Institute Inc, Cary, North Carolina, United States) will be used.

#### Qualitative Analysis

The qualitative interviews (individual interviews with people with dementia and relatives; focus groups with caregivers and dementia trainers) will be organized in the MAXQDA software program (VERBI GmbH, Berlin, Germany), and coded and analyzed by means of qualitative content analysis according to Schreier 2012 [59] by the Institute of Nursing Science.

## Results

As this is a study protocol with the study still in the intervention stage, no results are available as of yet. The study started in May 2019 and 18 participants with dementia (8 per group) have already finished the intervention. The study will end in spring 2020.

## Discussion

The overall aim of this study is to explore the effect of a SAR on psychosocial and physical outcomes of people with dementia living at home, including caregivers and dementia trainers. We hypothesize that the robot has a positive effect on the primary outcome motivation (stable or decreased apathy) of people with dementia.

### A Lack of Commercially Available and Tested Socially Assistive Robots

Research with SAR is a relatively young field [60], especially in people with dementia. A systematic review by Ienca et al [61] focusing on intelligent assistive technologies (including SAR) for people with dementia identified only 17/539 studies that included SAR. Furthermore, many studies were testing SAR in an (early) prototype stage [23,62]. Bedarf et al [62] identified in a review focusing on older people that, in general, only 6/107 robots were already commercially available. Buhtz et al [23] identified 3/13 SAR which are commercially available for older people in home care. In our study, the commercially available robot Pepper by SoftBank will be tested, which was refined for home care by our research team before this study according to the results of a qualitative study using a content analysis of interviews that included 80 participants (not yet published), and a first prototype test in home care with 12 participants (ClinicalTrials.gov Identifier: NCT03823066, results not yet published), including people with dementia, caregivers, and dementia trainers. The refined robot is illustrated in Figure 1. According to Alzheimer’s Disease International [1], in the absence of a medical solution for dementia, we need more research and innovation around care.

### Reasons of Institutionalization and Socially Assistive Robots as Nonpharmacological Home Care Intervention

Most often, research with robots like SAR is performed in laboratories and institutional settings such as nursing homes [13,15,63]. However, the home care setting is of high importance [1] because about 80% of people with dementia, especially in the early stages of the condition, receive care at home mainly through their relatives, with or without professional support [6,64,65]. Caregiver burden and the inability of informal caregivers to perform care on the person with dementia, beside neuropsychiatric symptoms/BPSD (especially apathy [66]), care dependency (in various ADL), mobility and cognition problems of the person with dementia, are some of the main reasons for institutionalization (eg, nursing home) [66-68]. Andel et al [69] stated that people with dementia are admitted earlier into a nursing home than people without such an illness. This situation shows that it is important to support caregivers of people with dementia in home care so that people with dementia can stay at home as long as possible. Our study includes cognitive and physical training by robot Pepper that belongs to the area of nonpharmacological interventions, where studies show that cognitive interventions may have a positive benefit for cognition and ADL, and physical training may improve or maintain ADL and may have a benefit for neuropsychiatric symptoms/BPSD [70-73].

### Relevance to Include Personal Views of People With Dementia

In our study, we included the personal experience of people with dementia because from the point of view of older care-dependent people, there is a scarcity of knowledge about the use of robots like SAR in real care situations [63]. It is highly recommended to include people with dementia and cognitive impairment in the design iteration cycles [74-77] because their feedback is very relevant for the appropriate and user-friendly development of novel technologies [76]. Furthermore, people with dementia are indeed able to learn to make use of robot technologies [77,78].

### Limitations of the Study

The study focuses only on people with mild to moderate dementia. Therefore, the results cannot be used for people with severe dementia. People with frontotemporal dementia were not included in the study because of known aggressive behavior. Therefore, we will not be able to obtain information as to whether people with this dementia type may benefit from a robot-based intervention. The study is performed only in home care, and results cannot be generalized to other settings, like nursing homes or hospitals.

Results and information of the ongoing study will be disseminated via our project homepage.

## Appendix

Multimedia Appendix 1 Measurements and time of data collection.

## Abbreviations

ADL: activities of daily living
AES: Apathy Evaluation Scale
BPSD: behavioral and psychological symptoms of dementia
NPI: Neuropsychiatric Inventory
SAR: socially assistive robot(s)
TUG: Timed UP and GO Test
