# Supplementary material for: The Effects of a Humanoid Socially Assistive Robot Versus Tablet Training on Psychosocial and Physical Outcomes of Persons With Dementia: Protocol for a Mixed Methods Study
Source: JMIR Res Protoc. 2020 Feb 5;9(2):e14927. doi: 10.2196/14927 (PMC7055795; doi:10.2196/14927)
Supplement: Multimedia Appendix 1 [file resprot_v9i2e14927_app1.docx]

| Point of measurement | | | Data collection method | Self-, proxy assessment, autonomous | Time of data collection | | | | | | | |
| --- | --- | --- | --- | --- | --- | --- | --- | --- | --- | --- | --- | --- |
|  | | |  |  | Enrollment | Week 0 | Week 1 | Week 2 | Week 3 | Week 0 | 5 months | 10 months |
|  | | |  |  |  |  |  |  |  |  |  |  |
| **Baseline measurement** | | | | | | | | | | | | |
|  | Sample characteristics (all participants) | | Questionnaire | Dementia: self and/or proxy; all other participants: self | X | X | — | — | — | X | — | — |
| **Primary outcomes** | | | | | | | | | | | | |
|  | **Persons with dementia** | | | | | | | | | | | |
|  |  | Motivation | Apathy Evaluation Scale (AES) | Self and proxy (by relatives) | — | X | — | — | — | X | — | — |
|  |  |  | Sensor data of the theratainment app | Autonomous | — | — | — | — | — | — | — | — |
|  | **Relatives** | | | | | | | | | | | |
|  |  | Care burden | Zarit Burden Interview | Self | — | X | — | — | — | X | — | — |
| **Secondary outcomes** | | | | | | | | | | | | |
|  | **Persons with dementia** | | | | | | | | | | | |
|  |  | Quality of life | Dementia Quality of Life | Self und proxy (by relatives) | — | X | — | — | — | X | — | — |
|  |  | Care dependency | Care Dependency Scale | Self und proxy (by relatives) | — | X | — | — | — | X | — | — |
|  |  | Mobility | Timed UP and GO Test (TUG) | Proxy (by clinical health care psychologist) | — | X | — | — | — | X | — | — |
|  |  |  | Sensor data of the eye camera of Pepper | Autonomous | — | — | — | — | — | — | — | — |
|  |  | Cognitive state | Montreal Cognitive Assessment | Proxy (by clinical health care psychologist) | — | X | — | — | — | X | — | — |
|  |  |  | Sensor data of the theratainment app, eye tracking | Autonomous | — | — | — | — | — | — | — | — |
|  |  | Depressive symptoms | The Geriatric Depression Scale-15 | Self | — | X | — | — | — | X | — | — |
|  |  | Behavioral problems | Neuropsychiatric Inventory (NPI) | Proxy (information of relative assessed by clinical health care psychologist) | — | X | — | — | — | X | — | — |
|  |  | Acceptance, usability, experience | Technology Usage Inventory (TUI) | Self | — | — | — | — | — | X | — | — |
|  |  |  | Observation | By nursing staff | — | — | X | — | X | — | — | — |
|  |  |  | Interviews | By nursing scientist | — | — | — | — | — | X | — | — |
|  | **Relatives** | | | | | | | | | | | |
|  |  | Quality of Life | World Health Organization Quality of Life Scale—BREF | Self | — | X | — | — | — | X | — | — |
|  |  | Depressive symptoms | Center for Epidemiological Studies Depression Scale | Self | — | X | — | — | — | X | — | — |
|  |  | Affect | Positive and Negative Affect Schedule | Self | — | X | — | — | — | X | — | — |
|  |  | Acceptance, usability, experience | TUI | Self | — | — | — | — | — | X | — | — |
|  |  |  | Interviews | By nursing scientist | — | — | — | — | — | X | — | — |
|  | **Dementia trainers** | | | | | | | | | | | |
|  |  | Acceptance, usability, experience | TUI | Self | — | X | — | — | — | — | X | X |
|  |  |  | Focus group | By nursing scientist | — | — | — | — | — | — | — | X |
|  | **Nursing staff** | | | | | | | | | | | |
|  |  | Acceptance, usability, experience | TUI | Self | — | X | — | — | — | — | X | X |
|  |  |  | Focus group | By nursing scientist | — | — | — | — | — | — | — | X |
